# N-Acetylcysteine Counteracts Immune Dysfunction and Autism-Related Behaviors in the Shank3b Mouse Model of Autism Spectrum Disorder

**DOI:** 10.3390/antiox13111390

**Published:** 2024-11-14

**Authors:** Luca Pangrazzi, Enrica Cerilli, Luigi Balasco, Ginevra Matilde Dall’O’, Gabriele Chelini, Anna Pastore, Birgit Weinberger, Yuri Bozzi

**Affiliations:** 1 Institute for Biomedical Aging Research, University of Innsbruck, Rennweg 10, 6020 Innsbruck, Austria; birgit.weinberger@uibk.ac.at; 2 CIMeC—Center for Mind/Brain Sciences, University of Trento, Piazza della Manifattura 1, 38068 Rovereto, Italy; enrica.cerilli@uu.se (E.C.); luigi.balasco@unitn.it (L.B.); ginevramdallo@gmail.com (G.M.D.); gabriele.chelini@unitn.it (G.C.); 3 Department of Life Sciences and Public Health, Universita’ Cattolica del Sacro Cuore, 00168 Rome, Italy; 4 CNR Neuroscience Institute, 56124 Pisa, Italy; 5 Metabolomics and Proteomics Unit, ‘Bambino Gesù’ Children’s Hospital, IRCCS, 00168 Rome, Italy; anna.pastore@opbg.net

**Keywords:** autism, inflammation, ROS, cerebellum, NAC

## Abstract

Autism spectrum disorder (ASD) includes a range of neurodevelopmental disabilities characterized by social interaction deficits, communication impairments, and repetitive behaviors. Previous studies have shown that pro-inflammatory conditions play a key role in ASD. Despite this, how oxidative stress and inflammation may contribute to ASD-related behaviors is still poorly understood. Here, we reported that increased levels of molecules related to inflammation are present in the cerebellum and peripheral blood (PB) of mice lacking Shank3b, an established model of syndromic ASD. In parallel, immune dysfunction was documented in the bone marrow (BM) and spleens of mutant mice. N-acetylcysteine (NAC) treatment rescued inflammation in the cerebellum and PB and impaired the production of pro-inflammatory molecules in the BM and spleen. In addition, social impairment was counteracted in NAC-treated *Shank3b^−/−^* animals. Taken together, our results provide clear evidence of the key role of cerebellar oxidative stress and inflammation in the establishment of ASD-related behaviors. Furthermore, our findings underscore the importance of considering ASD as a systemic disorder.

## 1. Introduction

Autism spectrum disorder (ASD) describes a group of neurodevelopmental disabilities associated with impairments in social interaction as well as social communication, repetitive behaviors, and interests [1]. Alongside the core symptoms, comorbidities including motor impairments, epilepsy, anxiety, depression, obsessive-compulsive disorder (OCD), and attention-deficit/hyperactivity disorder (ADHD) can be found in ASD [2,3]. The estimated worldwide prevalence of ASD is around 1/100 children, although it may be even higher in some countries [2,4]. ASD pathophysiology is very complex, as both genetic and environmental factors play important roles [5]. In the last 20 years, a consistent amount of work has been conducted to identify genes associated with ASD, and strategies of intervention have been proposed [6]. Despite this, effective medical treatments have not been identified yet.

Immune dysfunction and inflammation have been proposed as key contributors to the pathogenesis and severity of ASD [7]. In humans, increased levels of the pro-inflammatory molecules tumor necrosis factor (TNF), interleukin (IL)-1β, IL-6, IL-8, IL-12p40, and IL-17 were described in the plasma of children with ASD as compared with age-matched healthy controls [8,9]. Furthermore, cytokines IL-1β, IL-6, TNF, interferon (IFN)γ, and chemokine C-C motif ligand (CCL)-2) were overexpressed in the cerebrospinal fluid and brains of individuals with ASD [10,11]. Similarly, increased levels of pro-inflammatory molecules in brain areas and in the peripheral blood (PB) have been reported in ASD mouse models [7]. In particular, our recent work described pro-inflammatory dysfunction in the cerebellum and in the periphery of mice lacking the *Cntnap2* gene (*Cntnap2^−/−^* mice; [12]), a well-established model of syndromic ASD.

The accumulation of oxygen radicals, a condition commonly known as oxidative stress, can generally be found in the presence of inflammation. Increased ROS levels, paralleled by decreased antioxidant capacity, have been described in both ASD and mouse models [13,14,15,16]. Transcriptomic analyses performed in mice showed that genes coding for ROS-scavenging enzymes were less expressed in the brains of ASD model mice [7]. In *Cntnap2^−/−^* mice, we showed that systemic pro-inflammatory processes and ASD-related behaviors could be counteracted by the chronic administration of the antioxidant/anti-inflammatory molecule N-acetylcysteine (NAC; [12]). In studies performed in humans, NAC was shown to reduce irritability and stereotypic behaviors and improve social awareness [17]. Thus, strong evidence exists that a connection between oxidative stress and inflammation may play a determinant role in ASD.

Mice lacking the SH3 and multiple ankyrin repeat domains protein 3b (*Shank3b^−/^^−^* mice) represent a well-established mouse model of ASD, as they show deficits in social interaction, self-injurious repetitive grooming, and impaired locomotor activity [18,19]. In humans, SHANK3 mutations are responsible for the development of 22q13 deletion syndrome (Phelan–McDermid syndrome) and other non-syndromic forms of ASD [20].

In this study, we measured the expression of molecules related to inflammation in the cerebral cortex, hippocampus, cerebellum, and PB of *Shank3b^−/−^*, *Shank3b^+/−^*, and *Shank3b^+/+^* mice. In parallel, immune parameters were assessed in the bone marrow (BM) and spleen, lymphoid organs respectively involved in the production/maintenance of immune cells and in the generation of immune responses. Pro-inflammatory dysfunction was detected in the cerebella and PB of mutant animals. Furthermore, the differential expression of molecules involved in pro-inflammatory processes was additionally documented in the BM and spleen. Finally, NAC treatments could rescue social deficits, repetitive behaviors, and immune dysfunction in mutant mice. Altogether, this work strengthens the evidence that pro-inflammatory processes within the cerebellum may support ASD-related behaviors in mice.

## 2. Materials and Methods

### 2.1. Animals

Mice were housed following a 12 h light/dark cycle with food and water available *ad libitum*, taking care to minimize the animals pain and discomfort. Male and female *Shank3b^−/−^*, *Shank3b^+/−^*, and *Shank3b^+/+^* age-matched adult littermates (3–5 months old; weight 25–35 g) obtained from heterozygous mating were used. The numbers of mice used for each experiment are reported in the figure legends.

### 2.2. Tissue Harvesting

Brain samples used for RT-PCR were dissected and frozen in dry ice. Cerebella used for flow cytometry experiments were homogenized using a homogenizer immediately after dissection, and single-cell suspensions were prepared using a Falcon 70 μm cell strainer (Corning, New York, NY, USA). Approximately 200 μL of PB was harvested from each mouse and collected in a heparinized tube. PBMCs were isolated using a Ficoll–Hypaque density gradient (Sigma-Aldrich, St. Louis, MO, USA). BM cells were obtained after flushing femurs and tibias with PBS. Spleen samples were directly filtered through Falcon 70 µm cell strainers. After isolation, cerebella, PBMCs, BM, and spleen cells were washed once with RPMI 1640 (Sigma-Aldrich, St. Louis, MO, USA) and resuspended in complete medium (RPMI 1640 supplemented with 10% fetal calf serum, FCS; 100 U/mL penicillin and 100 μg/mL streptomycin; Sigma-Aldrich and Invitrogen, respectively).

### 2.3. RNA Isolation and Quantitative RT-PCR (qRT-PCR)

Total RNAs were extracted from the cerebral cortex, hippocampus, cerebellum, BM, and spleen samples of adult *Shank3b^−/−^*, *Shank3b^+/−^*, and *Shank3b^+/+^* mice using an RNeasy Plus Mini Kit (Qiagen, Hilden, Germany) and reverse transcribed to cDNA as reported in our previous work [12,21]. qRT-PCR was performed in a Bio-Rad C1000 Thermal Cycler, using the PowerUp™ SYBR™ Green Master Mix (Applied Biosystems, Waltham, MA, USA). Primers (Eurofins Genomics, Ebersberg, Germany) were designed on different exons to avoid the amplification of genomic DNA. The sequences of primers used for the study are shown in Table 1. CFX3 Manager 3.0 (Bio-Rad, Hercules, CA, USA) software was used to perform expression analyses. Mean cycle threshold (Ct) values from triplicate experiments were calculated for each gene of interest and the housekeeping gene β actin and then corrected for PCR efficiency and inter-run calibration. The expression of each mRNA of interest (normalized against β actin) was compared from triplicate experiments performed on RNA pools from 8–10 samples for each group. 

### 2.4. Flow Cytometry

Flow cytometry was used to quantify cytokine levels and immune cell populations in the cerebellum, PB, BM, and spleen. Immunofluorescence surface staining was performed by adding a panel of directly conjugated antibodies to freshly prepared cells. To assess the expression of cytokines, cells were incubated with 30 ng/mL phorbol 12-myristate 13-acetate (PMA) and 500 ng/mL ionomycin in the presence of 10 mg/mL brefeldin A (BFA) (all reagents from Sigma-Aldrich) for 4 h at 37 °C. After surface staining, cells were permeabilized using a Cytofix/Cytoperm kit (BD Biosciences, Franklin Lakes, NJ, USA) and incubated with intracellular antibodies. Labeled cells were measured using a LSR Fortessa (BD Biosciences) flow cytometer at the Institute for Biomedical Aging Research, University of Innsbruck. Data were analyzed using Flowjo software v10.8.1 (https://www.flowjo.com/). The antibodies used in the experiments are shown in Table 2.

### 2.5. NAC Treatment and Behavioral Tests

*Shank3b^−/−^*, *Shank3b^+/−^*, and *Shank3b^+/+^* mice were injected intraperitoneally with 50 mg/kg NAC (Sigma-Aldrich) resuspended in PBS or vehicle (PBS) for 28 consecutive days, as reported previously [12].

### 2.6. Behavioral Tests

Open field, rotarod, marble burying, and 3-chamber social tests were performed during the last 5 days of NAC treatment in *Shank3b^−/−^*, *Shank3b^+/−^*, and *Shank3b^+/+^* mice. In all behavioral tests, male and female mice were habituated and tested separately to avoid experimental noise.

### 2.7. Open Field Test

An open field test was performed to measure the motor activity of mice. Animals were placed in empty open-field arena (40 cm × 40 cm × 40 cm) and allowed to freely explore it for 20 min. Sessions were recorded by a camera located over the arena, and mice were automatically video tracked using the software EthoVisionXT (Noldus, https://www.noldus.com). Time spent moving and frequency in the center of the arena were analyzed.

### 2.8. Rotarod Test

Cerebellum-associated motor coordination was assessed using a rotarod test [22]. A habituation phase conducted at constant speed of 4 rpm was performed in the two days before the test. The experimental phase consisted of two trials in which the rotation speed was increased from 4 rpm to 64 rpm. Falling mice landed on a metallic platform that was connected to a timer showing the time spent on the rotating rod. The average time spent on the rod (latency to fall) during the second trial was measured in all experimental groups, and it was used as a quantitative indicator of the motor ability of the mice to stay balanced on the rotating accelerating rod.

### 2.9. Marble Burying Test

The tested mouse was placed in a cage containing bedding at a depth of 2 cm, and 20 black glass marbles were arranged on top of the bedding. The mouse was placed in the center of the cage for a 20 min exploration period under 15 lux illumination, and pictures were taken every 5 min. The number of marbles at least 50% covered by bedding were scored as buried.

### 2.10. Three-Chamber Social Test

The three-chamber social test was used to assess the social behaviors of mice [23]. The apparatus consisted of a plexiglass rectangular box (60 × 40 × 22 (h) cm, each chamber 20 × 40 × 22 (h) cm) with gray-colored walls and with removable panels separating the box into three chambers. On the four days before the beginning of the experimental phase, mice underwent a habituation phase in which they were placed in the three-chamber apparatus and allowed to freely explore it for 10 min. The experimental phase consisted of a 5 min habituation session, a 10 min exploration session, and the sociability test, in which one unfamiliar mouse was placed into a wire cylindrical cage (20 cm in height, 10 cm bottom-diameter, 1 cm bar spacing) in one of the upper corners of an external chamber. An identical empty wire cage was placed in the opposite external chamber. The tested mouse was allowed to freely interact with the unfamiliar mouse and with the wire cage for 10 min. The sociability index was calculated as the ratio between the time spent in the social chamber and the total time spent in the external chambers. Trials were recorded by an overhead camera placed over the three-chamber apparatus. Mice were automatically video tracked using the software EthoVisionXT (https://www.noldus.com).

### 2.11. Thiol Determination

Serum cysteinyl-glycine (Cys-Gly) levels were analyzed as previously reported [24].

### 2.12. Statistics

Statistical analyses were performed with GraphPad Prism 8.0 software, using one-way ANOVA and two-way ANOVA followed by Tukey’s post-hoc test, with the level of significance set at *p* < 0.05.

## 3. Results

### 3.1. Molecules Related to Inflammation Increase in the Cerebella of Mutant Shank3b Mice

**Figure 1:**
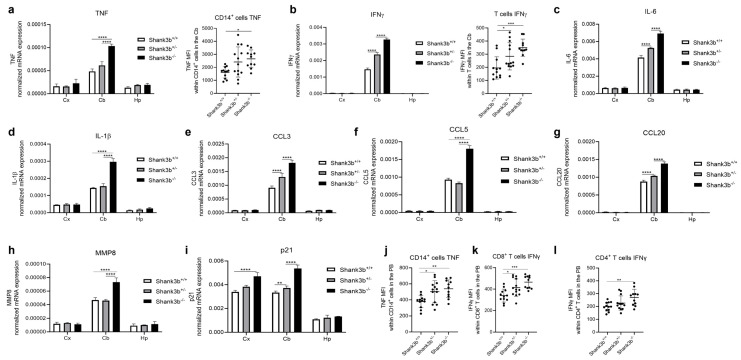
**Pro-inflammatory changes in the brain of *Shank3b^+/+^*, *Shank3b^+/−^*, and *Shank3b^−/−^* mice**. (**a**) TNF mRNA expression in the cerebral cortex (Cx), cerebellum (Cb), and hippocampus (Hp) of *Shank3b^+/+^*, *Shank3b^+/−^*, and *Shank3b^−/−^* mice measured using RT-qPCR and TNF expression at the protein level (shown as mean fluorescence intensity, MFI) within cerebellar CD14^+^ cells assessed using flow cytometry. (**b**) IFNγ mRNA expression in the Cx, Cb, and Hp and IFNγ levels within cerebellar T cells. mRNA expression of (**c**) IL-6, (**d**) IL-1β, (**e**) CCL3, (**f**) CCL5, (**g**) CCL20, (**h**) MMP8, and (**i**) p21 in the Cx, Cb, and Hp. mRNA expression was normalized against the housekeeping gene β-actin. (**j**) TNF MFI within CD14^+^ cells, IFNγ MFI within (**k**) CD8^+^ T cells and (**l**) CD4^+^ T cells in the PB of *Shank3b^+/+^*, *Shank3b^+/−^*, and *Shank3b^−/−^* mice. One-way ANOVA, Tukey’s post-hoc test. N = 8 per group (RT-qPCR); n = 10–12 per group (flow cytometry). * *p* < 0.05; ** *p* < 0.01; *** *p* < 0.001; **** *p* < 0.0001.

To assess whether inflammation may be present in the *Shank3b* model, the expression of key pro-inflammatory molecules was measured in the cerebral cortexes, cerebella, and hippocampi of *Shank3b*^+/+^, *Shank3b*^+/−^, and *Shank3b*^−/−^ mice (Figure 1). Overall, the levels of all molecules related to inflammation were increased in the cerebellum in comparison with those in other brain areas (Figure 1a–g). TNF expression within the cerebellum was the highest in *Shank3b*^−/−^ animals, while no differences were observed between *Shank3b*^+/−^ and *Shank3b*^+/+^ mice (Figure 1a). These results were confirmed using flow cytometry, which revealed increased TNF levels in CD14^+^ cerebellar cells from *Shank3b*^−/−^ animals. The gating strategy used for the flow cytometry experiments is shown in Supplementary Figure S1. IFNγ expression was detected in the cerebellum but not in the cerebral cortex or in the hippocampus (Figure 1b). Again, IFNγ levels were the highest in *Shank3b*^−/−^ and the lowest in *Shank3b*^+/+^ mice, while in this case, they were intermediate in the *Shank3b*^+/−^ group. The same results were described at the protein level when IFNγ expression was measured within T cells. Similar trends were observed for the pro-inflammatory molecules IL-6 and IL-1β, as their mRNAs were overexpressed in the cerebellum of *Shank3b*^−/−^ animals (Figure 1c,d). Chemokines CCL3, CCL5, and CCL20 control the chemotaxis of immune cells to sites of inflammation [25]. Again, mRNAs of all three molecules could be measured in the cerebellum only (Figure 1e–g). Also in this case, the expression of these chemokines was high in the *Shank3b*^−/−^ group, intermediate in *Shank3b*^+/−^ mice (at least for CCL3 and CCL20 mRNAs), and low in *Shank3b*^+/+^ controls. mRNA levels of metalloprotease (MMP) 8, known to support tissue damage and to play a determinant role in neuroinflammation [26], were increased within the cerebellum and again were the highest in *Shank3b*^−/−^ mice (Figure 1h). The inhibitor of cyclin-dependent kinases p21 (CDKN1A), activated during DNA damage response and additionally involved in cellular senescence [27], showed a similar mRNA expression in the cerebral cortex and in the cerebellum, while it was reduced in the hippocampus (Figure 1i). Interestingly, p21 mRNA levels within the cerebellum were low in the *Shank3b*^+/+^ group, intermediate in *Shank3b*^+/−^, and high in mutant animals. We next investigated whether pro-inflammatory changes may additionally be present in the peripheral blood (PB). Indeed, the expression of TNF in CD14^+^ cells and IFNγ within CD8^+^ and CD4^+^ T cells was increased in both *Shank3b*^+/−^ and *Shank3b*^−/−^ animals, in comparison with the control group (Figure 1j–l). Taken together, pro-inflammatory impairments are present in the cerebellum and in the PB of *Shank3b*^−/−^ and *Shank3b*^+/−^ mice.

### 3.2. Immune Dysfunction Is Present in the Bone Marrow and Spleen of Mutant Shank3b Mice

**Figure 2:**
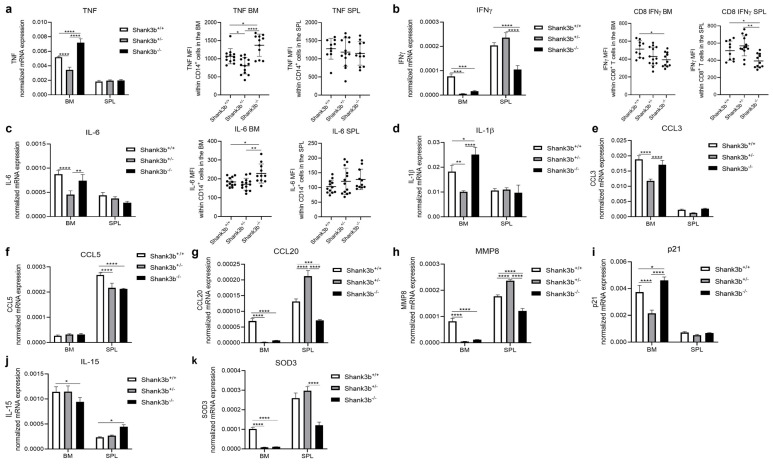
**Pro-inflammatory changes in the bone marrow and spleen of *Shank3b^+/+^*, *Shank3b^+/−^*, and *Shank3b^−/−^* mice**. (**a**) TNF mRNA expression in the bone marrow (BM) and spleen (SPL) of *Shank3b^+/+^*, *Shank3b^+/−^* and *Shank3b^−/−^* mice measured using RT-qPCR and TNF expression at the protein level (shown as mean fluorescence intensity, MFI) within CD14^+^ cells assessed using flow cytometry. (**b**) IFNγ mRNA expression and IFNγ levels within CD8^+^ T cells. (**c**) IL-6 mRNA expression and IL-6 levels within CD14^+^ cells. mRNA expression of (**d**) IL-1β, (**e**) CCL3, (**f**) CCL5, (**g**) CCL20, (**h**) MMP8, (**i**) p21, (**j**) IL-15, and (**k**) SOD3 in the BM and SPL. mRNA expression was normalized against the housekeeping gene β-actin. One-way ANOVA, Tukey’s post-hoc test. n = 8 per group (RT-qPCR); n = 10–12 per group (flow cytometry). * *p* < 0.05; ** *p* < 0.01; *** *p* < 0.001; **** *p* < 0.0001.

As a next step, we assessed whether immune system dysfunction may additionally be observed in the bone marrow (BM) and spleens of *Shank3b*^+/−^ and *Shank3b*^−/−^ mice (Figure 2). In both organs, the expression of pro-inflammatory molecules varied consistently across genotypes. Within the BM, although TNF was overexpressed in *Shank3b*^−/−^ animals in comparison to the control group, the levels of this molecule were reduced in *Shank3b*^+/−^ mice (Figure 2a). No differences were observed in the spleen. These results were confirmed at the protein level using flow cytometry, in which TNF expression was assessed in CD14^+^ cells, a population including macrophages and monocytes. No differences were found within CD8^+^ and CD4^+^ T cells in either BM or spleen tissue (Figure 2a and Supplementary Figure S2a–d). Surprisingly, IFNγ mRNA levels in the BM were reduced in both *Shank3b*^+/−^ and *Shank3b*^−/−^ mice and in the spleens of *Shank3b*^−/−^ animals when compared with those in the *Shank3b*^+/+^ group (Figure 2b). Similar results were obtained when IFNγ expression was measured within CD8^+^ T cells and in CD4^+^ T cells, although no differences between *Shank3b*^+/+^ and *Shank3b*^+/−^ were observed (Figure 2b and Supplementary Figure S2e,f). Furthermore, comparable IL-6 levels were detected in *Shank3b*^+/+^ and *Shank3b*^−/−^ mice in the BM, while the expression of this cytokine was again reduced in *Shank3b*^+/−^ mice (Figure 2c). Despite this, increased IL-6 levels in *Shank3b*^−/−^ mice and similar expression between the other two groups were observed within BM CD14^+^ cells. In addition, no differences were identified in the spleen. IL-1β mRNA expression was again increased in the BM of *Shank3b*^−/−^ mice and decreased in *Shank3b*^+/−^ mice in comparison with that in the control group, while no differences were found in the spleen (Figure 2d). We next investigated the mRNA expression of chemokines CCL3, CCL5, and CCL20 in the BM and in the spleen (Figure 2e–g). Interestingly, when *Shank3b*^+/+^ and *Shank3b*^−/−^ animals were compared, no differences for CCL3 and CCL8 and decreased CCL20 mRNA levels were observed in the BM of mutant mice. In the spleen, reduced CCL5 and CCL20 mRNA levels were found in *Shank3b*^−/−^ animals when compared with controls. In parallel, decreased CCL3 and CCL20 mRNA levels in the BM, as well as reduced CCL5 mRNA and increased CCL20 mRNA levels in the spleen, were described in *Shank3b*^+/−^ animals compared with values the *Shank3b*^+/+^ group. Thus, these results suggest that the migratory capacity of immune cells in the BM and in the spleen may be different between *Shank3b*^+/+^, *Shank3b*^+/−^, and *Shank3b*^−/−^ mice. Similarly, MMP8 mRNA levels were decreased in the BM of *Shank3b*^+/−^ and *Shank3b*^−/−^ animals, reduced in the spleen of *Shank3b*^−/−^, and increased in the *Shank3b*^+/−^ group (Figure 2i). Furthermore, p21 mRNA expression in the BM was upregulated in *Shank3b*^−/−^ and downregulated in *Shank3b*^−/+^ mice when compared with control animals, while no differences were present in the spleen (Figure 2i). IL-15 is known to play a determinant role in the maintenance of immune cells in the BM and in the establishment of immunological memory in the spleen [28,29]. mRNA levels of this cytokine in *Shank3b*^−/−^ mice were found to be reduced in the BM and increased in the spleen as compared with the other two groups. Finally, we observed that mRNA expression of the antioxidant enzyme SOD3 was decreased in the BM and spleens of *Shank3b*^−/−^ animals and in the BM of *Shank3b*^+/−^ mice (Figure 2k). Taken together, these results show that the levels of molecules related to inflammation in the BM and spleen change in *Shank3b*^+/−^ and *Shank3b*^−/−^ mice. In *Shank3b*^−/−^ mice, while pro-inflammatory processes are induced in the BM, they may be inhibited in the spleen. Furthermore, these impairments may be accompanied by oxidative stress.

### 3.3. N-Acetyl-Cysteine Improves ASD-Related Behaviors in Shank3b^−/−^ Mice

**Figure 3:**
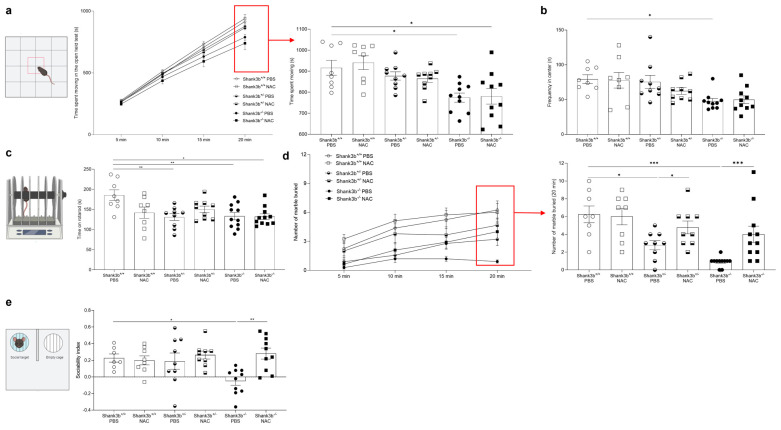
**Behavioral tests in *Shank3b^+/+^*, *Shank3b^+/−^*, and *Shank3b^−/−^* mice treated with NAC**. Time spent moving (s) (**a**) and frequency in the center (**b**) in the open field test in NAC-treated *Shank3b^+/+^*, *Shank3b^+/−^*, and *Shank3b^−/−^* mice and PBS-treated control animals. (**c**) Time on the rotarod (latency to fall, s) in the rotarod test. (**d**) Number of marbles buried in the marble burying test. (**e**) Sociability index (time in the sniffing zone of mouse chamber—time in the sniffing zone of empty chamber)/total time in the sniffing zones) in the three-chamber test. n = 8 (*Shank3b^+/+^*, PBS), n = 8 (*Shank3b^+/+^*, NAC), n = 9 (*Shank3b^+/−^*, PBS), n = 9 (*Shank3b^+/−^*, NAC), n = 9–11 (*Shank3b^−/−^*, PBS), n = 10 (*Shank3b^−/−^*, NAC). Two-way ANOVA, Tukey’s post-hoc test. * *p* < 0.05; ** *p* < 0.01, *** *p* < 0.001.

In our previous work on mice lacking the ASD-relevant gene *Cntnap2*, we showed that N-acetylcysteine (NAC) may act as anti-inflammatory molecule within the cerebellum and in the periphery [12]. To assess whether ASD-related behaviors may be improved in the *Shank3b* model, NAC was intraperitoneally injected once a day for 28 days into *Shank3b*^+/+^, *Shank3b*^+/−^, and *Shank3b*^−/−^ mice. At the end of the treatment, behavioral tests were performed on NAC- and PBS-treated mice to assess motor (open field and rotarod test), repetitive (marble burying test), and social behaviors (three-chamber social test) (Figure 3). As previously described [18,19], PBS-treated *Shank3b*^−/−^ mice were hypoactive and spent less time moving in the open-field arena when compared to control mice (Figure 3a). These differences increased over time, and they were particularly evident after 20 min of testing. In addition, *Shank3b*^−/−^ mice spent less time in the central part of the arena compared to the *Shank3b*^+/+^ mice, suggesting that anxiety-like behaviors may be present in these mice (Figure 3b). Despite this, no differences across genotypes were found after the administration of NAC. Furthermore, motor coordination was measured in NAC- and PBS-treated animals using a rotarod test (Figure 3c). Reduced time on the rotarod was found in both PBS-treated *Shank3b*^+/−^ and *Shank3b*^−/−^ mice in comparison with *Shank3b*^+/+^ controls, but no changes were observed after NAC administration. Next, a marble burying test was performed to investigate repetitive behaviors (Figure 3d). Again, differences between groups became evident over time, and they were quantified at the end of the test (20 min). When the PBS-treated groups were compared, the number of buried marbles decreased in *Shank3b*^+/−^ and was the lowest in *Shank3b*^−/−^ animals in comparison with *Shank3b*^+/+^ mice. Interestingly, a partial rescue in the phenotype was observed in both *Shank3b*^+/−^ and *Shank3b*^−/−^ animals after the injection of NAC (Figure 3d). We finally assessed social behavior in all experimental groups using the three-chamber social test (Figure 3e). As expected, PBS-treated *Shank3b*^−/−^ mice showed a reduced sociability index compared to *Shank3b*^+/+^ control mice. Importantly, social impairments were completely rescued in *Shank3b*^−/−^ mice administered with NAC. No differences were observed between *Shank3b*^+/−^ and *Shank3b*^+/+^ mice in either the PBS- or NAC-treated groups. Taken together, these results show that NAC treatment could completely rescue sociability in *Shank3b*^−/−^ mice.

### 3.4. NAC Reduces Pro-Inflammatory Impairments in the Cerebellum and Peripheral Blood of Shank3b^−/−^ Mice

**Figure 4:**
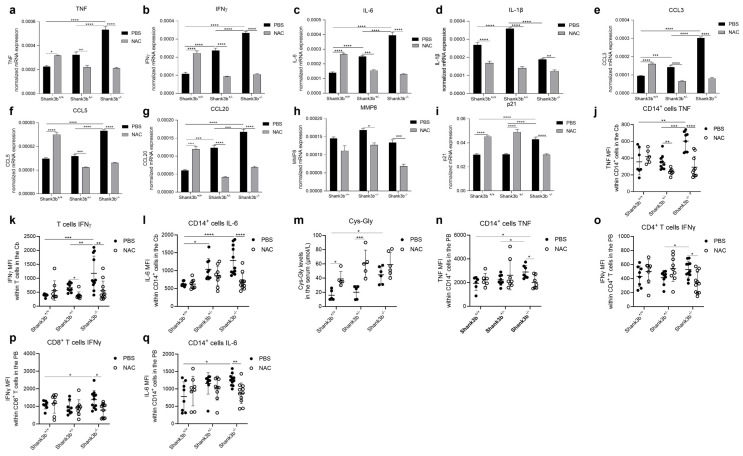
**Pro-inflammatory molecules in the cerebellum of *Shank3b^+/+^*, *Shank3b^+/−^*, and *Shank3b^−/−^* mice treated with NAC**. mRNA expression of (**a**) TNF, (**b**) IFNγ, (**c**) IL-6, (**d**) IL-1β, (**e**) CCL3, (**f**) CCL5, (**g**) CCL20, (**h**) MMP8, and (**i**) p21 in the cerebellum of NAC-treated *Shank3b^+/+^*, *Shank3b^+/−^*, and *Shank3b^−/−^* mice and PBS-treated control animals assessed using RT-qPCR. mRNA expression was normalized against the housekeeping gene β-actin. n=8 in each group. Mean fluorescence intensity (MFI) of (**j**) TNF within CD14^+^ cells, (**k**) IFNγ within T cells, and (**l**) IL-6 within CD14^+^ cells in the cerebellum measured using flow cytometry. (**m**) Cys-Gly levels in the serum of PBS- and NAC-treated animals. (**n**) TNF within CD14^+^ cells, (**o**) IFNγ within CD4^+^ T cells, (**p**) IFNγ within CD8^+^ T cells, and (**q**) IL-6 within CD14^+^ cells in the peripheral blood (PB). n = 8 (*Shank3b^+/+^*, PBS), n = 8 (*Shank3b^+/+^*, NAC), n = 9 (*Shank3b^+/−^*, PBS), n = 9 (*Shank3b^+/−^*, NAC), n = 9–11 (*Shank3b^−/−^*, PBS), n = 10 (*Shank3b^−/−^*, NAC); for (m) N = 5–6 in each group. Two-way ANOVA, Tukey’s post-hoc test. * *p* < 0.05; ** *p* < 0.01, *** *p* < 0.001, **** *p* < 0.0001.

To investigate whether improvements in ASD-related behaviors may be paralleled by decreased inflammation, pro-inflammatory molecules were quantified in the cerebellum of *Shank3b*^+/+^, *Shank3b*^+/−^, and *Shank3b*^−/−^ mice treated either with PBS or NAC (Figure 4). A decreased expression of TNF, IFNγ, IL-6, and IL-1β mRNAs was detected in both *Shank3b*^+/−^ and *Shank3b*^−/−^ mice treated with NAC compared to the controls (Figure 4a–d). As previously observed in *Cntnap2* mutant mice [12], the levels of these molecules (with the exception of IL-1β) were increased after NAC treatment in *Shank3b*^+/+^ mice. Similar trends were observed for CCL3, CCL5, and CCL20 chemokines, which showed reduced mRNA expression in the cerebellum in *Shank3b*^+/−^ and *Shank3b*^−/−^ mice and increased levels in the *Shank3b*^+/+^ mice treated with NAC compared to their respective PBS-treated controls (Figure 4e–g). Furthermore, MMP8 levels were again downregulated in the NAC-treated *Shank3b*^+/−^ and *Shank3b*^−/−^ groups, while no differences were found for *Shank3b*^+/+^ mice (Figure 4h). In addition, while p21 expression was increased in NAC-treated *Shank3b*^+/+^ and *Shank3b*^+/−^ animals, the levels of this molecule were decreased in the *Shank3b*^−/−^ group compared to their respective PBS-treated controls (Figure 4i). In line with these observations, TNF, IFNγ, and IL-6 protein levels (respectively within CD14^+^ cells, T cells, and again CD14^+^ cells) were decreased in NAC-treated *Shank3b*^+/−^ and *Shank3b*^−/−^ mice (Figure 4 j–l). No differences were detected for *Shank3b*^+/+^ mice. These results indicate that the expression of molecules related to inflammation/damage was reduced in the cerebellum of *Shank3b*^−/−^ mice treated with NAC.

We next assessed whether NAC treatments may additionally target oxidative stress and inflammation in the PB. The plasma levels of cysteinyl-glycine (Cys-Gly) in *Shank3b*^+/+^, *Shank3b*^+/−^, and *Shank3b*^−/−^ mice increased after the administration of NAC, suggesting that glutathione synthesis may be induced in these animals (Figure 4m). When TNF expression was measured in CD14^+^ cells within peripheral blood mononuclear cells (PBMCs), decreased levels of this cytokine were found within monocytes (CD14^+^ cells) and CD4^+^ T cells (but not in CD8^+^ T cells) in NAC-treated *Shank3b*^−/−^ mice compared with those in PBS-treated *Shank3b*^−/−^ animals (Figure 4n and Supplementary Figure S3a,b). Similarly, IFNγ expression within CD4^+^ and CD8^+^ T cells, as well as IL-6 expression within CD14^+^ cells and CD4^+^ and CD8^+^ T cells, was reduced in NAC-treated *Shank3b*^−/−^ mice (Figure 4n–q and Supplementary Figure S3c,d). No differences were observed for *Shank3b*^+/+^ and *Shank3b*^+/−^ animals. Taken together, these findings indicate that NAC treatment rescued pro-inflammatory dysfunction in both the cerebella and PB of *Shank3b*^−/−^ animals.

### 3.5. NAC Counteracts Immune Dysfunction in the Bone Marrow of Shank3b^−/−^ Mice

**Figure 5:**
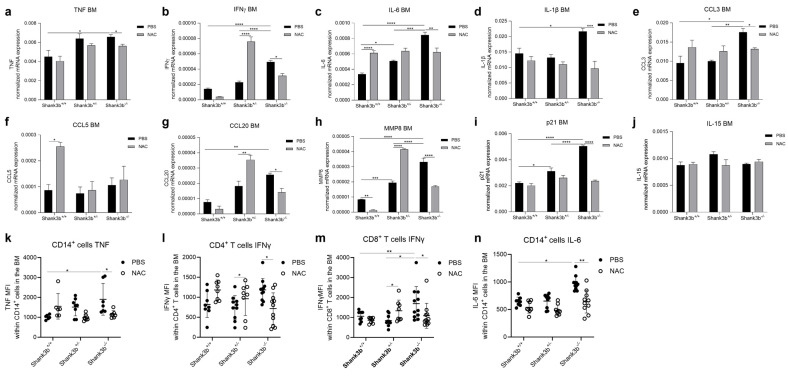
**Pro-inflammatory molecules in the bone marrow of *Shank3b^+/+^*, *Shank3b^+/−^*, and *Shank3b^−/−^* mice treated with NAC**. mRNA expression of (**a**) TNF, (**b**) IFNγ, (**c**) IL-6, (**d**) IL-1β, (**e**) CCL3, (**f**) CCL5, (**g**) CCL20, (**h**) MMP8, (**i**) p21, and (**j**) IL-15 in the bone marrow (BM) of NAC-treated *Shank3b^+/+^*, *Shank3b^+/−^*, and *Shank3b^−/−^* mice and PBS-treated control animals assessed using RT-qPCR. mRNA expression was normalized against the housekeeping gene β-actin. n = 8 in each group. Mean fluorescence intensity (MFI) of (**k**) TNF within CD14^+^ cells, (**l**) IFNγ within CD4^+^ T cells, (**m**) IFNγ within CD8^+^ T cells, and (**n**) IL-6 within CD14^+^ cells in the BM measured using flow cytometry. n = 8 (*Shank3b^+/+^*, PBS), n = 8 (*Shank3b^+/+^*, NAC), n = 9 (*Shank3b^+/−^*, PBS), n = 9 (*Shank3b^+/−^*, NAC), n = 9–11 (*Shank3b^−/−^*, PBS), n = 10 (*Shank3b^−/−^*, NAC); for (**m**) N = 5–6 in each group. Two-way ANOVA, Tukey’s post-hoc test. * *p* < 0.05; ** *p* < 0.01, *** *p* < 0.001, **** *p* < 0.0001.

As the imbalanced production of pro-inflammatory cytokines was additionally observed in the BM of *Shank3b*^−/−^ mice, the expression of molecules related to inflammation was assessed within the BM of PBS- and NAC-treated mutant and *Shank3b*^+/+^ mice (Figure 5). The levels of all TNF, IFNγ, IL-6, and IL-1β in *Shank3b*^−/−^ mice were reduced after NAC administration (Figure 5a–d). The situation was completely different for the *Shank3b*^+/−^ group, in which NAC treatment increased IFNγ mRNA levels but had no effect on the expression of the other molecules in comparison with their respective controls. Furthermore, increased IL-6 mRNA levels and no differences in the other molecules were found in *Shank3b*^+/+^ animals. Similar results were observed when the mRNA expression of CCL3, CCL5, and CCL20 chemokines was assessed (Figure 5e–g). Following NAC treatment, CCL3 and CCL20 mRNA levels decreased in *Shank3b*^−/−^ mice, CCL20 increased in *Shank3b*^+/−^ mice, and CCL5 increased in *Shank3b*^+/+^ animals compared to their respective PBS-treated controls. Furthermore, NAC reduced MMP8 expression in the *Shank3b*^+/+^ and *Shank3b*^−/−^ groups, while increased levels were found in NAC-treated *Shank3b*^+/−^ mice. In parallel, similarly to what was observed in the cerebellum, p21 mRNA expression was decreased in NAC-treated *Shank3b*^−/−^ mice, while no differences were found in the other groups. In addition, the levels of memory T cell-survival factor IL-15 did not vary between the groups (Figure 5j). Similar results were obtained when the expression of pro-inflammatory cytokines was measured at the protein level (Figure 5k–n). Indeed, the reduced expression of TNF within CD14^+^ cells, IFNγ in CD4^+^ and CD8^+^ T cells, and IL-6 within CD14^+^ cells was observed in NAC-treated *Shank3b*^−/−^ mice compared with that in the PBS-treated mice. Increased IFNγ levels in CD4^+^ and CD8^+^ T cells were present in NAC-treated *Shank3b*^+/−^ mice, while no differences were detected in the *Shank3b*^+/+^ group. Taken together, these results indicate that NAC counteracts immune dysfunction in the BM of *Shank3b*^−/−^ mice.

### 3.6. NAC Boosts Pro-Inflammatory Processes in the Spleen of Shank3b^−/−^ Mice

**Figure 6:**
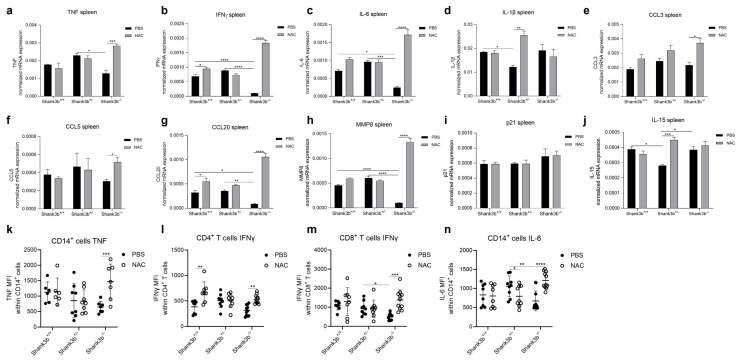
**Pro-inflammatory molecules in the spleens of *Shank3b^+/+^*, *Shank3b^+/−^*, and *Shank3b^−/−^* mice treated with NAC**. mRNA expression of (**a**) TNF, (**b**) IFNγ, (**c**) IL-6, (**d**) IL-1β, (**e**) CCL3, (**f**) CCL5, (**g**) CCL20, (**h**) MMP8, (**i**) p21, and (**j**) IL-15 in the spleens of NAC-treated *Shank3b^+/+^*, *Shank3b^+/−^*, and *Shank3b^−/−^* mice and PBS-treated control animals assessed using RT-qPCR. mRNA expression was normalized against the housekeeping gene β-actin. n=8 in each group. Mean fluorescence intensity (MFI) of (**k**) TNF within CD14^+^ cells, (**l**) IFNγ within CD4^+^ T cells, (**m**) IFNγ within CD8^+^ T cells, and (**n**) IL-6 within CD14^+^ cells in the spleen measured using flow cytometry. n = 8 (*Shank3b^+/+^*, PBS), n = 8 (*Shank3b^+/+^*, NAC), n = 9 (*Shank3b^+/−^*, PBS), n = 9 (*Shank3b^+/−^*, NAC), n = 9–11 (*Shank3b^−/−^*, PBS), n = 10 (*Shank3b^−/−^*, NAC). Two-way ANOVA, Tukey’s post-hoc test. * *p* < 0.05; ** *p* < 0.01, *** *p* < 0.001, **** *p* < 0.0001.

Reduced pro-inflammatory responses were found in the spleens of *Shank3b*^−/−^ mice in basal conditions (Figure 2). We therefore investigated whether NAC treatments may affect the production of pro-inflammatory molecules in spleen cells from *Shank3b*^+/+^, *Shank3b*^+/−^, and *Shank3b*^−/−^ mice (Figure 6). Interestingly, the mRNA expression of TNF, IFNγ, and IL-6 in *Shank3b*^−/−^ animals strongly increased after NAC administration (Figure 6a–c). No differences were observed in *Shank3b*^+/+^ and *Shank3b*^+/−^ mice. In parallel, IL-1β levels (which were reduced in PBS-treated *Shank3b*^+/−^ mice compared with PBS-treated *Shank3b*^+/+^ controls) increased in the *Shank3b*^+/−^ group after NAC treatment (Figure 6d). No differences were found in *Shank3b*^−/−^ animals. Furthermore CCL3, CCL5, CCL20, and MMP8 mRNAs were overexpressed in NAC-treated *Shank3b*^−/−^ mice compared with their respective PBS-treated controls (Figure 6e–g). Increased CCL20 mRNA expression was additionally documented for *Shank3b*^+/+^ mice, while no differences could be detected for *Shank3b*^+/−^ animals. Furthermore, p21 mRNA expression in the spleen was not affected by NAC (Figure 6j). IL-15 mRNA expression increased after NAC treatment in *Shank3b*^+/−^ mice (Figure 6j). No changes were observed in the other groups. We then assessed the expression of the pro-inflammatory molecules TNF, IFNγ, and IL-6 using flow cytometry (Figure 6k–n). Similar to the previous results, the levels of TNF within CD14^+^ cells, IFNγ in CD4^+^ and CD8^+^ T cells, and IL-6 in CD14^+^ cells increased in NAC-treated *Shank3b*^−/−^ mice (Figure 6l). In summary, NAC administration increases the production of pro-inflammatory molecules in the spleen of *Shank3b*^−/−^ animals, suggesting that NAC may play an important role in supporting immune responses in mutant mice.

## 4. Discussion

Several recent studies have described increased levels of pro-inflammatory molecules and oxidative stress in children with ASD and in mouse models [7,8,9,10,11,12,13,14,15,16]). As recently proposed, inflammation may impair the maturation of vulnerable neurons, therefore supporting the onset of ASD [30]. Despite this, the origin of these pro-inflammatory conditions is still unknown.

In this work, we investigated the expression of molecules related to inflammation in the cerebral cortex, hippocampus, and cerebellum in a *Shank3b* mouse model of ASD. The disruption of *Shank3* has been associated with core neurodevelopmental and neurobehavioral deficits in 22q13 deletion syndrome (Phelan–McDermid syndrome), a syndromic form of ASD [20]. Previous work from our and other laboratories showed that mice lacking the *Shank3b* variant (*Shank3b^−^*^/*−*^ mice) showed pronounced ASD-related behaviors, including social impairment, self-injurious repetitive grooming, and sensory differences [18,19]. Despite this, whether pro-inflammatory conditions may be present in these mice was never assessed before. Because in clinical conditions, *Shank3* mutations are generally present in heterozygosis, our analysis was further extended to *Shank3b^+^*^/*−*^ animals. In line with our recent observations in *Cntnap2^−/−^* mice (another mouse model of syndromic ASD; [12,31]), the overexpression of pro-inflammatory molecules was specifically restricted to the cerebellum in *Shank3b^−/−^* mice (Figure 1). Indeed, *Shank3b* is highly expressed in the cerebellum [32]. Overall, an intermediate level of inflammation was present in *Shank3b^+/−^* animals, suggesting that *Shank3b* gene expression influences pro-inflammatory responses within the cerebellum. In particular, all key pro-inflammatory cytokines (TNF, IFNγ, IL-6, IL-1β) and molecules involved in immune cell migration (CCL3, CCL5, and CCL20) were upregulated in the *Shank3b^−/−^* cerebellum. While TNF was increased in CD14^+^ cells, innate immune cells that include macrophages and microglia cells [33,34], IFNγ was overexpressed in T cells. CCL3, CCL5, and CCL20 are known to be induced in the presence of pro-inflammatory molecules, as they regulate the migration of immune cells to sites of inflammation [25]. Altogether, these three chemokines play a determinant role in neuroinflammation, as CCL3 regulates the functions of macrophages and astrocytes [35], while CCL5 and CCL20 mainly control T-cell migration [36,37]. Within the cerebellum, chemokines support the pro-inflammatory profile of microglia [38]. In addition, to promote the migration of immune cells, chemokines induce the expression of MMPs, including MMP8, a molecule involved in tissue remodeling [39]. Indeed, MMP8 within the cerebellum was highly expressed in *Shank3b^−/−^* mice. Furthermore, the cell cycle inhibitor p21, typically induced in the presence of DNA damage and considered a marker of cellular senescence [27,40], was additionally overexpressed in *Shank3b^−/−^* animals. Importantly, the mRNA expression of most of the molecules (IFNγ, IL-6, CCL3, CCL20, and p21) in *Shank3b^+/−^* mice was intermediate between *Shank3b^+/+^* and *Shank3b^−/−^* mice. In parallel, pro-inflammatory impairments were additionally detected in the PB in both *Shank3b^+/−^* and *Shank3b^−/−^* animals.

Unexpectedly, lower mRNA levels of pro-inflammatory molecules were detected in the BM of *Shank3b^+/−^* mice compared to those in control *Shank3b^+/+^* mice. The situation was even more complex for *Shank3b^−/−^* mice, which showed increased levels of TNF, IL-1β, and p21 and decreased expression of IFNγ, CCL20, and MMP8. We can therefore speculate that in the BM, the functionality of the immune system may be impaired in *Shank3b^+/−^* mice, although some changes observed at the mRNA level may be at least partially “buffered” at the protein level (i.e., IFNγ and IL-6). Immune dysfunction may additionally be present in *Shank3b^−/−^* mice, although in this case, it may be compensated for by the systemic increase in pro-inflammatory molecules. Fewer differences were found in the spleen, in which the most significant differences were represented by decreased IFNγ, CCL5, CCL20, and MMP8 mRNA levels in *Shank3b^−/−^* animals. In addition, the antioxidant enzyme SOD3 was shown to be decreased in mutant mice in both the BM and spleen.

We therefore administered the antioxidant/anti-inflammatory molecule NAC, which in our previous study was shown to target cerebellar as well as systemic inflammation in *Cntnap2^−/−^* mice [12]. Differently from *Cntnap2^−/−^* mice, no rescuing effects were observed on motor deficits in *Shank3b^−/−^* animals, as detected by the open field and rotarod tests. This apparent inconsistency may be explained by a key difference between the *Cntnap2* and *Shank3b* models, as *Cntnap2^−/−^* mice are hyperactive, while *Shank3b^−/−^* mice are hypoactive. Thus, NAC may be more effective in counteracting hyperactivity rather than hypoactivity, as previously observed [41]. In line with previous studies [42], *Shank3b^+/−^* and *Shank3b^−/−^* mice buried a lower number of marbles in the marble burying test compared to control mice. Interestingly, NAC restored this phenotype, indicating that it may at least partially rescue hypoactivity in *Shank3b^−/−^* mice. Most importantly, NAC administration was able to rescue sociability deficits in *Shank3b^−/−^* animals. These results were accompanied by decreased levels of pro-inflammatory molecules in the cerebellum, as all molecules related to inflammation were reduced in both *Shank3b^+/−^* and *Shank3b^−/−^* mice. Similarly to our previous observations in the *Cntnap2* model [12], an overall increase in the expression of pro-inflammatory molecules was observed in *Shank3b^+/+^* mice treated with NAC; this might be caused by “antioxidative stress”, a stressful condition supported by the excessive elimination of oxygen radicals [43,44]. It is however worth noting that despite this pro-inflammatory activity of NAC, we did not observe social impairment in the NAC-treated *Shank3b^+/+^* group.

Similar results were detected in the PB. In this context, increased Cys-Gly levels (indicating glutathione synthesis) were found in the serum of all NAC-treated groups. Thus, in all animals, NAC may effectively be converted into glutathione, although the effects of the drug may also be achieved in a glutathione-independent manner [45]. In the PB, all pro-inflammatory molecules of interest were decreased in *Shank3b^−/−^* mice, while only some of them were reduced in *Shank3b^+/−^* animals. Intriguingly, treatments by themselves increased levels of inflammation in the BM of *Shank3b^+/−^* and *Shank3b^−/−^* mice, as in animals treated with vehicle, the expression of all key pro-inflammatory cytokines was increased when compared with that in *Shank3b^+/−^* and *Shank3b^−/−^* mice in basal conditions (Figure 1 and Figure 2). This might be caused by a reduced capability of mutant mice to counteract stress. Overall, NAC administration partially boosted immune functions in the BM of *Shank3b^+/−^* mice, as IFNγ was increased in *Shank3b^+/−^* animals, while some molecules (IL-6 and CCL5) were additionally induced in the *Shank3b^+/+^* group. In parallel, the levels of most pro-inflammatory molecules were reduced in NAC-treated *Shank3b^−/−^* mice when compared with those in their PBS-treated counterparts. This was accompanied by decreased levels of p21. Thus, in *Shank3b^−/−^* animals, the administration of NAC may “balance” the levels of pro-inflammatory molecules and counteract damage.

The spleen represents the largest lymphoid organ, and the production of pro-inflammatory molecules by immune cells within the spleen exerts a vital role in immunity. Thus, reduced levels of cytokines in this organ are generally associated with immune dysfunction [46]. Interestingly, our results suggest that immune dysfunction observed in *Shank3b^−/−^* animals could be counteracted by NAC, as the production of all key pro-inflammatory molecules was strongly increased. Some changes were additionally documented for *Shank3b^+/+^* and *Shank3b^+/−^* mice. Importantly, in our study, we assessed chronic inflammation, i.e., the levels of pro-inflammatory molecules present in the brain and in the periphery without exposure to external antigens. Future research must investigate inflammatory responses after triggering the immune system of mutant and control mice, for example with model antigens.

In summary, this study contributes to strengthening the hypothesis that pro-inflammatory dysfunction within the cerebellum may be associated with ASD-related behaviors. In addition, we showed that immune dysfunction extends to the PB, BM, and spleen, and it can be counteracted after the administration of NAC. Although these differences were clearer in *Shank3b^−/−^* mice, a certain degree of immune dysfunction was additionally present in *Shank3b^+/−^* animals, and NAC was also effective in this group. This represents an important observation that may be considered when developing novel strategies for intervention in clinical settings. Furthermore, this study underlines the importance of addressing ASD as a “systemic disorder” and not as a condition affecting the brain only.

## Figures and Tables

**Table 1:** Sequences of the primers used for this study.

Target Gene	Forward Primer (5′–3′)	Reverse Primer (5′–3′)
*TNF* 21926	CAAAATTCGAGTGACAAGCC	TGTCTTTGAGATCCATGCCG
*IFNγ* 15978	CCCTATGGAGATGACGGAGA	CTGTCTGCTGGTGGAGTTCA
*IL-6* 16193	GCCTTCTTGGGACTGATGCT	GACAGGTCTGTTGGGAGTGG
*IL-1β* 16176	ACGGACCCCAAAAGATGAAG	TTCTCCACAGCCACAATGAG
*CCL3* 20302	TGAAACCAGCAGCCTTTGCT	AGGCATTCAGTTCCAGGTCAGTG
*CCL5* 20304	AGAATACATCAACTATTTGGAGA	CCTTGCATCTGAAATTTTAATGA
*CCL20* 20297	CTTGCTTTGGCATGGGTACT	TCAGCGCACACAGATTTTCT
*MMP8* 17394	AATCCTTGCCCATGCCTTTCAACC	CCAAATTCATGAGCAGCCACGAGA
*p21(* *CDKN1A) 12575*	GACAAGAGGCCCAGTACTTC	GCTTGGAGTGATAGAAATCTGTC
*IL-15* 16168	ATCCATCTCGTGCTACTTGTGTT	CATCTATCCAGTTGGCCTCTGTTT
*SOD3 20657*	CCAGCTTCGACCTAGCAGACA	CAGCGTGGCTGATGGTTGTA
*β actin* 11461	GGCTGTATTCCCCTCCATCG	CCAGTTGGTAACAATGCCATGT

**Table 2:** FACS antibodies used for this study.

Antigen	Fluorochrome	Company	Clone
CD3	APC-Vio770	Miltenyi; Cologne, Germany	REA641
CD8	PerCp	Biolegend, San Diego, CA, USA	53–6.7
CD4	VioGreen	Miltenyi Cologne, Germany	REA1211
CD14	Pecy7	Biolegend, San Diego, CA, USA	Sa14–2
TNF	PE	Biolegend, San Diego, CA, USA	MP6-XT22
IFNγ	FITC	Miltenyi Cologne, Germany	REA638
IL-6	APC	Biolegend, San Diego, CA, USA	MP5-20F3

## Data Availability

All data are available upon request from the correspondent authors.

## Supplementary Materials

The following supporting information can be downloaded at: https://www.mdpi.com/article/10.3390/antiox13111390/s1, Figure S1. Gating strategy used for the flow cytometry experiments. Figure S2. Pro-inflammatory molecules within T cells in the bone marrow and spleen of *Shank3b^+/+^*, *Shank3b^+/−^* and *Shank3b^−/−^* mice. Figure S3. Pro-inflammatory molecules in the PB of *Shank3b^+/+^*, *Shank3b^+/−^* and *Shank3b^−/−^* mice treated with NAC.

## References

[1] American Psychiatric Association (2013). Diagnostic and Statistical Manual of Mental Disorders.

[2] Zeidan J., Fombonne E., Scorah J., Ibrahim A., Durkin M.S., Saxena S., Yusuf A., Shih A., Elsabbagh M. (2022). Global prevalence of autism: A systematic review update. Autism Res..

[3] Khachadourian V., Mahjani B., Sandin S., Kolevzon A., Buxbaum J.D., Reichenberg A., Janecka M. (2023). Comorbidities in autism spectrum disorder and their etiologies. Transl. Psychiatry.

[4] Maenner M.J. (2023). Prevalence and Characteristics of Autism Spectrum Disorder Among Children Aged 8 Years—Autism and Developmental Disabilities Monitoring Network, 11 Sites, United States, 2020. MMWR. Surveill. Summ..

[5] Chaste P., Leboyer M. (2012). Autism risk factors: Genes, environment, and gene-environment interactions. Dialog. Clin. Neurosci..

[6] Fu J.M., Satterstrom F.K., Peng M., Brand H., Collins R.L., Dong S., Wamsley B., Klei L., Wang L., Hao S.P. (2022). Rare coding variation provides insight into the genetic architecture and phenotypic context of autism. Nat. Genet..

[7] Pangrazzi L., Balasco L., Bozzi Y. (2020). Oxidative stress and immune system dysfunction in autism spectrum disorders. Int. J. Mol. Sci..

[8] Ashwood P., Krakowiak P., Hertz-Picciotto I., Hansen R., Pessah I., Van de Water J. (2011). Elevated plasma cytokines in autism spectrum disorders provide evidence of immune dysfunction and are associated with impaired behavioral outcome. Brain Behav. Immun..

[9] Jácome M.C.I., Chacòn L.M.M., Cuesta H.V., Rizo C.M., Santiesteban M.W., Hernandez L.R., García E.N., Fraguela M.E.G., Verdecia C.I.F., Hurtado Y.V. (2016). Peripheral inflammatory markers contributing to comorbidities in autism. Behav. Sci..

[10] Li X., Chauhan A., Sheikh A.M., Patil S., Chauhan V., Li X.-M., Ji L., Brown T., Malik M. (2009). Elevated immune response in the brain of autistic patients. J. Neuroimmunol..

[11] Vargas D.L., Nascimbene C., Krishnan C., Zimmerman A.W., Pardo C.A. (2005). Neuroglial activation and neuroinflammation in the brain of patients with autism. Ann. Neurol..

[12] Pangrazzi L., Cerilli E., Balasco L., Tobia C., Dall’O’ G.M., Chelini G., Perini S., Filosi M., Ravizza T., Vezzani A. (2023). The interplay between oxidative stress and inflammation supports autistic-related behaviors in mice. Biorxiv.

[13] James S.J., Melnyk S., Jernigan S., Cleves M.A., Halsted C.H., Wong D.H., Cutler P., Bock K., Boris M., Bradstreet J.J. (2006). Metabolic endophenotype and related genotypes are associated with oxidative stress in children with autism. Am. J. Med Genet. Part B Neuropsychiatr. Genet..

[14] Chen L., Shi X.-J., Liu H., Mao X., Gui L.-N., Wang H., Cheng Y. (2021). Oxidative stress marker aberrations in children with autism spectrum disorder: A systematic review and meta-analysis of 87 studies (N = 9109). Transl. Psychiatry.

[15] Rose S., Melnyk S., Pavliv O., Bai S., Nick T.G., Frye R.E., James S.J. (2012). Evidence of oxidative damage and inflammation associated with low glutathione redox status in the autism brain. Transl. Psychiatry.

[16] Liu X., Lin J., Zhang H., Khan N.U., Zhang J., Tang X., Cao X., Shen L. (2022). Oxidative stress in autism spectrum disorder—Current progress of mechanisms and biomarkers. Front. Psychiatry.

[17] Lee T.-M., Lee K.-M., Lee C.-Y., Lee H.-C., Tam K.-W., Loh E.-W. (2021). Effectiveness of *N*-acetylcysteine in autism spectrum disorders: A meta-analysis of randomized controlled trials. Aust. N. Z. J. Psychiatry.

[18] Peça J., Feliciano C., Ting J.T., Wang W., Wells M.F., Venkatraman T.N., Lascola C.D., Fu Z., Feng G. (2011). Shank3 mutant mice display autistic-like behaviours and striatal dysfunction. Nature.

[19] Balasco L., Pagani M., Pangrazzi L., Chelini G., Chama A.G.C., Shlosman E., Mattioni L., Galbusera A., Iurilli G., Provenzano G. (2022). Abnormal Whisker-Dependent Behaviors and Altered Cortico-Hippocampal Connectivity in *Shank3b*−/− Mice. Cereb. Cortex.

[20] Phelan K., McDermid H. (2012). The 22q13.3 Deletion Syndrome (Phelan-McDermid Syndrome). Mol. Syndr..

[21] Pangrazzi L., Genovesi S., Balasco L., Cerilli E., Robol C., Zunino G., Piazza S., Provenzano G., Bozzi Y. (2022). Immune dysfunction in the cerebellum of mice lacking the autism candidate gene Engrailed 2. J. Neuroimmunol..

[22] Caston J., Jones N., Stelz T. (1995). Role of preoperative and postoperative sensorimotor training on restoration of the equilibrium behavior in adult mice following cerebellectomy. Neurobiol. Learn. Mem..

[23] Moy S.S., Nadler J.J., Perez A., Barbaro R.P., Johns J.M., Magnuson T.R., Piven J., Crawley J.N. (2004). Sociability and preference for social novelty in five inbred strains: An approach to assess autistic-like behavior in mice. Genes Brain Behav..

[24] Pastore A., Panera N., Mosca A., Caccamo R., Camanni D., Crudele A., De Stefanis C., Alterio A., Di Giovamberardino G., De Vito R. (2021). Changes in total homocysteine and glutathione levels after laparoscopic sleeve gastrectomy in children with metabolic-associated fatty liver disease. Obes. Surg..

[25] Hughes C.E., Nibbs R.J.B. (2018). A guide to chemokines and their receptors. FEBS J..

[26] Lee E.J., Han J.E., Woo M.S., Shin J.A., Park E.M., Kang J.L., Moon P.G., Baek M.C., Son W.S., Ko Y.T. (2014). Matrix met-alloproteinase-8 plays a pivotal role in neuroinflammation by modulating TNF-α activation. J. Immunol..

[27] Jurk D., Wang C., Miwa S., Maddick M., Korolchuk V., Tsolou A., Gonos E.S., Thrasivoulou C., Saffrey M.J., Cameron K. (2012). Postmitotic neurons develop a p21-dependent senescence-like phenotype driven by a DNA damage response. Aging Cell.

[28] Cui G., Hara T., Simmons S., Wagatsuma K., Abe A., Miyachi H., Kitano S., Ishii M., Tani-Ichi S., Ikuta K. (2014). Characterization of the IL-15 niche in primary and secondary lymphoid organs in vivo. Proc. Natl. Acad. Sci. USA.

[29] Meryk A., Grasse M., Balasco L., Kapferer W., Grubeck-Loebenstein B., Pangrazzi L. (2020). Antioxidants N-Acetylcysteine and Vitamin C Improve T Cell Commitment to Memory and Long-Term Maintenance of Immunological Memory in Old Mice. Antioxidants.

[30] Ament S.A., Cortes-Gutierrez M., Herb B.R., Mocci E., Colantuoni C., McCarthy M.M. (2023). A single-cell genomic atlas for matura-tion of the human cerebellum during early childhood. Sci. Transl. Med..

[31] Peñagarikano O., Abrahams B.S., Herman E.I., Winden K.D., Gdalyahu A., Dong H., Sonnenblick L.I., Gruver R., Almajano J., Bragin A. (2011). Absence of CNTNAP2 leads to epilepsy, neuronal migration abnormalities, and core autism-related deficits. Cell.

[32] Wang X., Xu Q., Bey A.L., Lee Y., Jiang Y.-H. (2014). Transcriptional and functional complexity of Shank3 provides a molecular framework to understand the phenotypic heterogeneity of SHANK3 causing autism and Shank3 mutant mice. Mol. Autism.

[33] Janova H., Böttcher C., Holtman I.R., Regen T., van Rossum D., Götz A., Ernst A., Fritsche C., Gertig U., Saiepour N. (2016). CD14 is a key organizer of microglial responses to CNS infection and injury. Glia.

[34] Beschorner R., Schluesener H.J., Gözalan F., Meyermann R., Schwab J.M. (2002). Infiltrating CD14+ monocytes and expression of CD14 by activated parenchymal microglia/macrophages contribute to the pool of CD14+ cells in ischemic brain lesions. J. Neuroimmunol..

[35] Banisor I., Leist T.P., Kalman B. (2005). Involvement of β-chemokines in the development of inflammatory demyelination. J. Neuroinflammation.

[36] Hieshima K., Imai T., Opdenakker G., Van Damme J., Kusuda J., Tei H., Sakaki Y., Takatsuki K., Miura R., Yoshie O. (1997). Molecular cloning of a novel human CC chemokine liver and activation-regulated chemokine (LARC) expressed in liv-er. Chemotactic activity for lymphocytes and gene localization on chromosome 2. J. Biol. Chem..

[37] Murooka T.T., Rahbar R., Platanias L.C., Fish E.N. (2008). CCL5-mediated T-cell chemotaxis involves the initiation of mRNA translation through mTOR/4E-BP1. Blood.

[38] Škuljec J., Sun H., Pul R., Bénardais K., Ragancokova D., Moharregh-Khiabani D., Kotsiari A., Trebst C., Stangel M. (2011). CCL5 induces a pro-inflammatory profile in microglia in vitro. Cell. Immunol..

[39] Page-McCaw A., Ewald A.J., Werb Z. (2007). Matrix metalloproteinases and the regulation of tissue remodelling. Nat. Rev. Mol. Cell Biol..

[40] González-Gualda E., Baker A.G., Fruk L., Muñoz-Espín D. (2021). A guide to assessing cellular senescence in vitro and in vivo. FEBS J..

[41] Lai C.-C., Baskaran R., Tsao C.-Y., Tuan L.-H., Siow P.-F., Palani M., Lee L.J.-H., Liu C.-M., Hwu H.-G., Lee L.-J. (2022). Chronic N-Acetylcysteine Treatment Prevents Amphetamine-Induced Hyperactivity in Heterozygous *Disc1* Mutant Mice, a Putative Prodromal Schizophrenia Animal Model. Int. J. Mol. Sci..

[42] Dhamne S.C., Silverman J.L., Super C.E., Lammers S.H.T., Hameed M.Q., Modi M.E., Copping N.A., Pride M.C., Smith D.G., Rotenberg A. (2017). Replicable in vivo physiological and behavioral phenotypes of the Shank3B null mutant mouse model of autism. Mol. Autism.

[43] Villanueva C., Kross R.D. (2012). Antioxidant-Induced Stress. Int. J. Mol. Sci..

[44] Dündar Y., Aslan R. (2000). Antioxidative stress. East. J. Med..

[45] Kannan N., Nguyen L.V., Makarem M., Dong Y., Shih K., Eirew P., Raouf A., Emerman J.T., Eaves C.J. (2014). Glutathione-dependent and -independent oxidative stress-control mechanisms distinguish normal human mammary epithelial cell subsets. Proc. Natl. Acad. Sci. USA.

[46] Lewis S.M., Williams A., Eisenbarth S.C. (2019). Structure and function of the immune system in the spleen. Sci. Immunol..

