# Supplementary material for: N-Acetylcysteine Counteracts Immune Dysfunction and Autism-Related Behaviors in the Shank3b Mouse Model of Autism Spectrum Disorder
Source: Antioxidants (Basel). 2024 Nov 14;13(11):1390. doi: 10.3390/antiox13111390 (PMC11590982; doi:10.3390/antiox13111390)
Supplement: Supplementary file 1 [file antioxidants-13-01390-s001.zip › antioxidants-3244650-supplementary.pptx]

## Slide 1
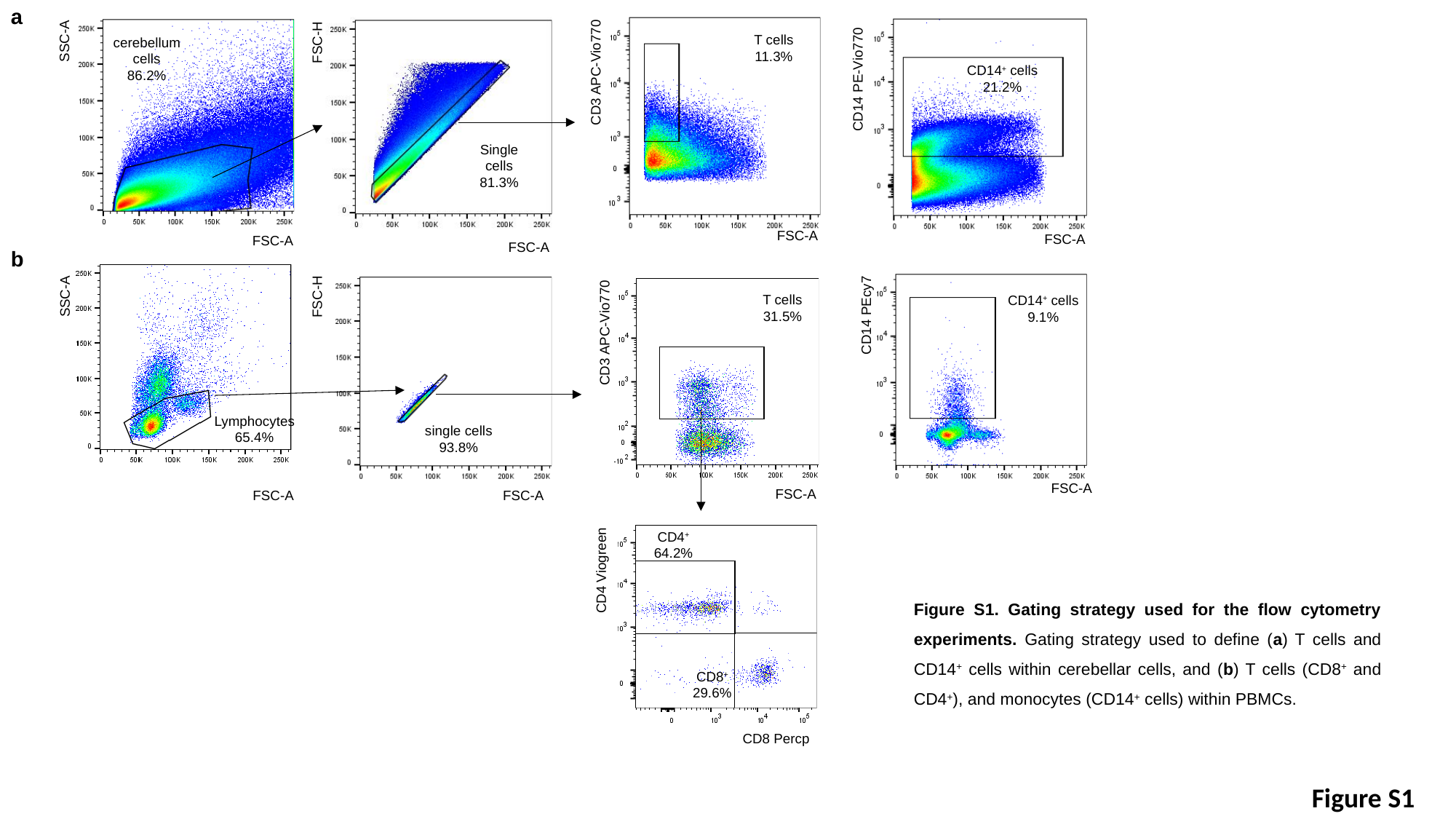

SSC-A
cerebellum cells
86.2%
FSC-A
FSC-H
Single cells
81.3%
FSC-A
T cells
11.3%
CD3 APC-Vio770
FSC-A
CD14+ cells
21.2%
CD14 PE-Vio770
FSC-A
a
b
Lymphocytes
65.4%
CD14 PEcy7
FSC-A
FSC-H
FSC-A
T cells
31.5%
CD3 APC-Vio770
FSC-A
CD4 Viogreen
CD8 Percp
CD4+
64.2%
CD8+
29.6%
single cells
93.8%
SSC-A
FSC-A
CD14+ cells
9.1%
Figure S1. Gating strategy used for the flow cytometry experiments. Gating strategy used to define (a) T cells and CD14+ cells within cerebellar cells, and (b) T cells (CD8+ and CD4+), and monocytes (CD14+ cells) within PBMCs.
Figure S1

## Slide 2
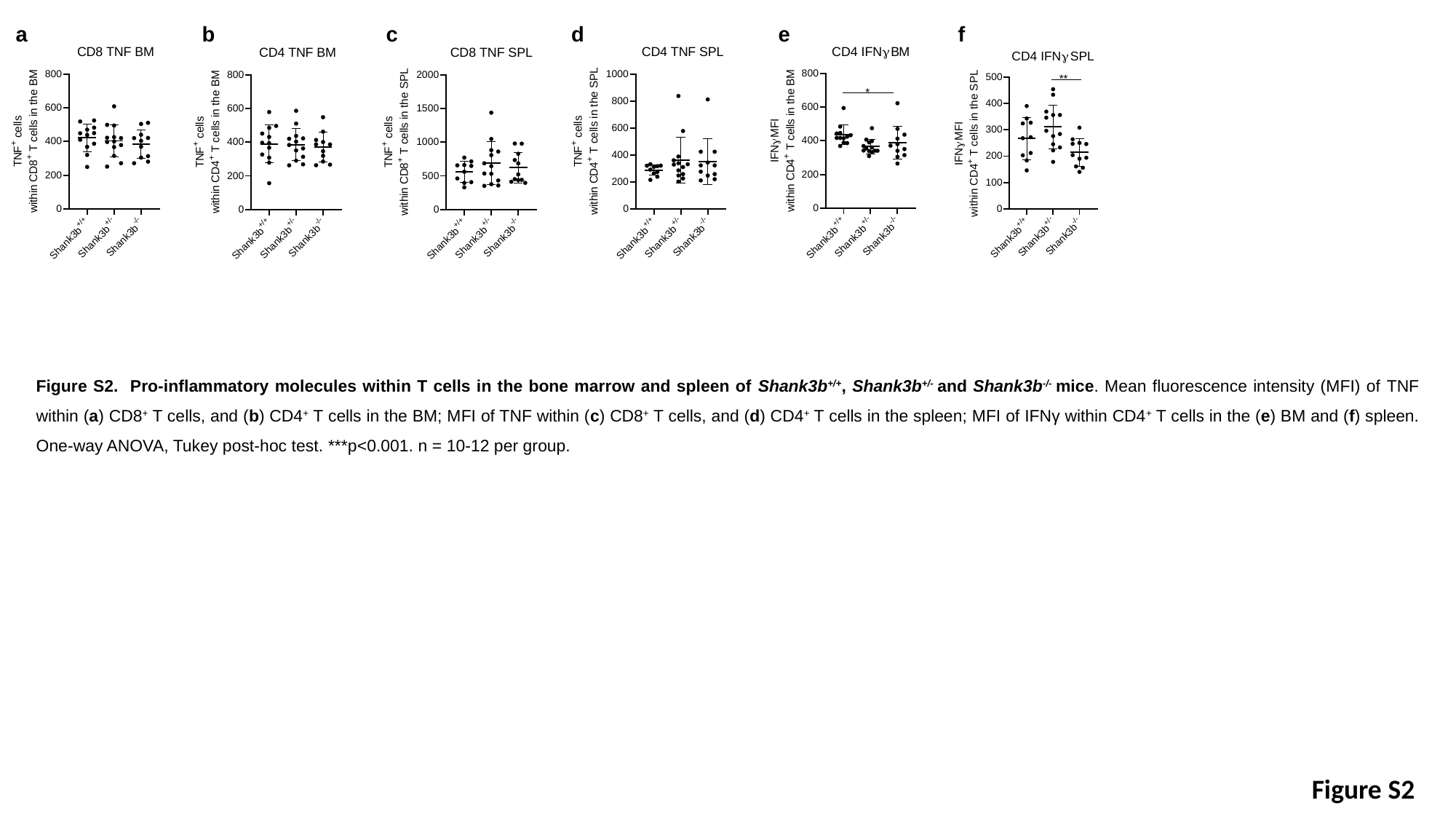

a
b
c
d
e
f
Figure S2
Figure S2. Pro-inflammatory molecules within T cells in the bone marrow and spleen of Shank3b+/+, Shank3b+/- and Shank3b-/- mice. Mean fluorescence intensity (MFI) of TNF within (a) CD8+ T cells, and (b) CD4+ T cells in the BM; MFI of TNF within (c) CD8+ T cells, and (d) CD4+ T cells in the spleen; MFI of IFNγ within CD4+ T cells in the (e) BM and (f) spleen. One-way ANOVA, Tukey post-hoc test. ***p<0.001. n = 10-12 per group.

## Slide 3
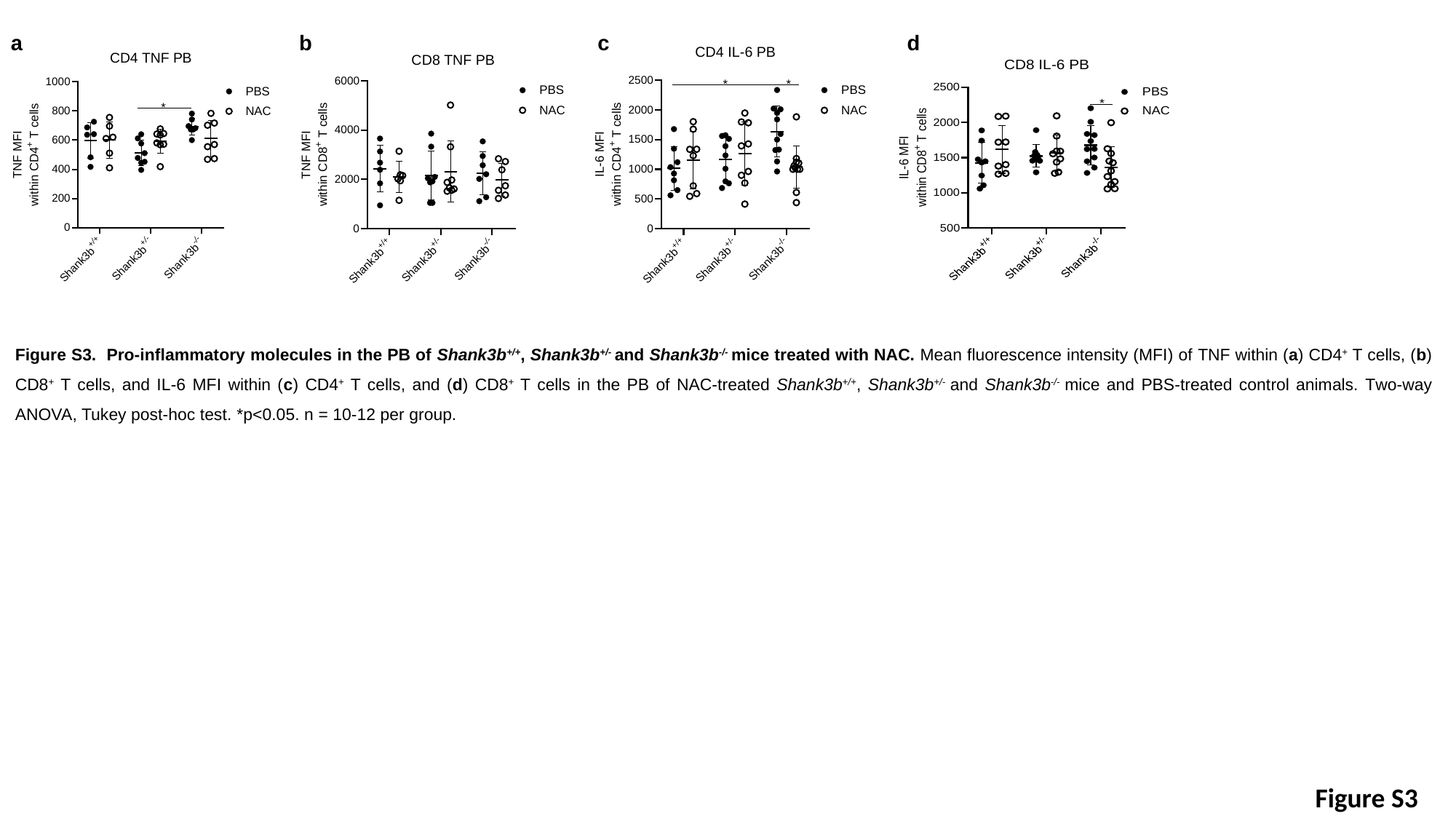

a
b
c
d
Figure S3
Figure S3. Pro-inflammatory molecules in the PB of Shank3b+/+, Shank3b+/- and Shank3b-/- mice treated with NAC. Mean fluorescence intensity (MFI) of TNF within (a) CD4+ T cells, (b) CD8+ T cells, and IL-6 MFI within (c) CD4+ T cells, and (d) CD8+ T cells in the PB of NAC-treated Shank3b+/+, Shank3b+/- and Shank3b-/- mice and PBS-treated control animals. Two-way ANOVA, Tukey post-hoc test. *p<0.05. n = 10-12 per group.
